# Optical Detection Techniques for Biomedical Sensing: A Review of Printed Circuit Board (PCB)-Based Lab-on-Chip Systems

**DOI:** 10.3390/mi16050564

**Published:** 2025-05-08

**Authors:** Francisco Perdigones, Pablo Giménez-Gómez, Xavier Muñoz-Berbel, Carmen Aracil

**Affiliations:** 1 Electronic Engineering Department, Higher Technical School of Engineering, University of Seville, 41092 Seville, Spain; aracilc@us.es; 2 Department of Materials and Environmental Chemistry, Stockholm University, 106 91 Stockholm, Sweden; pablo.gimenez-gomez@su.se; 3 Institut de Microelectrònica de Barcelona (IMB-CNM, CSIC), Universitat Autònoma de Barcelona, 08193 Cerdanyola del Vallès, Spain; xavier.munoz@imb-cnm.csic.es

**Keywords:** Lab-on-PCB, printed circuit boards, biomedical applications, fluorescence, absorbance, chemiluminescence

## Abstract

Lab on Printed Circuit Boards (Lab-on-PCB) technology has emerged as a promising platform, offering miniaturization, integration, and cost-effective fabrication for a wide range of sensing applications. This review explores the most common optical detection techniques implemented on printed circuit boards (PCBs), including absorbance, fluorescence, and chemiluminescence, discussing their working principles, advantages, and limitations in the context of PCB-based sensing. Additionally, evanescent wave generation is considered as an alternative optical approach with benefits for specific applications. Elements such as excitation sources, photodetectors, and the distinguishing characteristics of each method are analyzed to provide a comprehensive, but concise, overview of the field. Emphasis is placed on how the PCB platform influences the performance, sensitivity, and feasibility of these detection methods, highlighting relevant design considerations. This work aims to provide a solid foundation for researchers interested in optical sensing within this technology, serving as a reference for future developments and applications in PCB-based optical detection.

## 1. Introduction

The use of printed circuit board (PCB) substrates for the development of microfluidic devices has gained significant attention in research [1]. These devices, known as lab-on-PCB (LoP), are considered a subset of the lab-on-a-chip (LoC) systems [2]. Lab-on-a-chip systems are miniaturized to approximately a few centimeters, with microfluidic circuits being in the micrometer to millimeter range. This miniaturization enables high levels of integration, combining several laboratory functions on a chip with the aforementioned dimensions, which improves efficiency. These devices enable fast processes, enabling mixing, reaction, and analysis, which are the main functions for applications such as point-of-care diagnostics and real-time monitoring.

Therefore, this article will discuss examples of both systems, beginning with general examples of Lab-on-Chip systems, and subsequently focusing on Lab-on-PCB systems, which constitute the main subject of this review. The PCB substrates offer interesting advantages for biomedical applications [3], including the integration of electronics and microfluidics with sensors and actuators on a unified platform, their well-known commercial reliability, and low production costs, as we will study in this work. Notably, lab-on-PCB systems are capable of performing several laboratory functions, such as micromixing, sensing, biochemical reactions, and heating in the same platform. However, some required characteristics and configurations of particular methods pose challenges for PCB technology, as will be explained in Section 6.

Although many optical systems have been developed for biomedical applications, such as standard pin and avalanche photodiodes, phototransistors, CCDs (charge-coupled devices), photomultipliers (PMTs), and CMOS (complementary metal–oxide–semiconductor) sensors [4,5,6], those specifically designed for LoCs will be discussed first, including the following: a device for assessing cell viability, fabricated using polydimethylsiloxane (PDMS), that incorporates a photodiode and an LED (light-emitting diode) as its optical components [6] and a device for pH measurement via absorbance, constructed from glass and lithium niobate, which employs a photodiode and a laser beam [7]. These devices share the crucial characteristic for optical detection, transparency, which enables efficient light transmission and interaction. Many more examples with similar principles can be found in the literature [8,9]. Notably, the transition from silicon to glass as a primary material for lab-on-a-chip devices was largely motivated by the transparency of glass, enabling the integration of optical methods and enhancing their functionality on these platforms, making them more adaptable and efficient for biomedical applications. These examples highlight the important role that material selection plays in the implementation of certain detection methods.

However, while traditional optical methods such as absorbance, fluorescence, and SPR (surface plasmon resonance), are commonly used in microfluidic platforms, miniaturizing them for low-cost, portable devices remains challenging. A key challenge in designing fully integrated optical LoC devices is incorporating multiple components, including light sources, microfluidic networks, and photodetectors. The integration of these components can be achieved either monolithically, embedding all functionalities on a single chip, or through a modular approach, where the components are distributed on multiple chips. Modular systems offer cost advantages and enable the reuse of components. However, optimizing the interfaces between the various subsystems remains a demanding technical task [10]. The advantages of integrating the detection system into LoC platforms [11] are so significant that they have driven the emergence of the field of optofluidics. This interdisciplinary area merges optical sensing with fluidic manipulation to achieve highly efficient and automated detection processes [10,12]. These technologies highlight the importance of integration and versatility and the potential of optical sensors to boost the development of robust lab-on-a-chip platforms capable of real-time, sensitive, and specific detection for a wide range of biomedical applications. As research in integrated optics progresses, optical biosensors are maybe the most important component of advanced LoC systems, transforming fields such as healthcare, environmental monitoring, and biotechnological research.

The current state of the art showcases the application of lab-on-PCB systems in many biomedical fields, including detecting cell viability [13], molecular diagnosis [14], organotypic cultures [15], virus genetic detection [16], and electrolytes detection [17]. All of these applications require detection systems, which are usually electrochemical and optical. Regarding optical methods specifically integrated into lab-on-PCB systems, many examples can be found in the literature on the use of these technologies in absorbance-, fluorescence-, and chemiluminescence-based implementations. However, some methods cannot fully utilize the inherent characteristics of printed circuit boards directly and efficiently. In fact, in this study, PCBs’ limitations and compatibility with some phenomena will be discussed.

Since optical methods are the core of this work, we will briefly mention that electrochemical sensors are quite common in PCB-based biomedical applications [18], and several interesting examples can be found in the literature, as follows: an electrochemical biosensor array for quantitative polymerase chain reaction [19]; a low-cost electrochemical biosensor for rapid bacterial detection [20]; the multiplex PCB-based electrochemical detection of cancer biomarkers using an MLPA (multiplex ligation-dependent probe amplification)-barcode approach [21]; and a lab-on-PCB system that adapts colorimetric methods, such as enzyme-linked immunosorbent assay (ELISA), into electrochemical approaches [22], to name a few. In addition, focusing on flexible PCB for wearable applications, there are notable examples available, including a flexible electrochemical glucose sensor with a composite nanostructured surface for the working electrode [23] or for the detection of SARS-CoV-2 [24]. These sensors can be integrated thanks to the possibilities offered by the copper layers of the PCB and, among other processes, the electroless nickel immersion gold ENIG process [25].

Throughout this work, we will focus on the study of different optical detection systems, first analyzing their specific characteristics, followed by the examination of practical implementations in Lab-on-a-Chip platforms, and concluding with selected examples developed in Lab-on-PCB systems. In addition, various strategies for optical sensors will be analyzed, studying its compatibility with lab-on-PCB technology, such as surface plasmon resonance. Furthermore, the advantages and disadvantages of commercially available printed circuit boards, including both rigid and flexible substrates, will be examined in order to provide a general overview and encourage further developments in this field.

## 2. Absorbance

Absorbance is the most intuitive and direct method in optical detection systems [26,27]. This procedure is based on the Beer–Lambert law, which states that the absorbance of a substance is directly proportional to its concentration and the optical path length of the light passing through the sample [28]. First, the sample containing the substance to be measured, such as a protein, dye, or other light-interacting compound that interacts with light, is prepared. This sample is then placed in an appropriate medium, such as a quartz or glass cuvette, and exposed to a monochromatic light source, with the wavelength selected according to the specific analysis to be performed.

**Figure 1:**
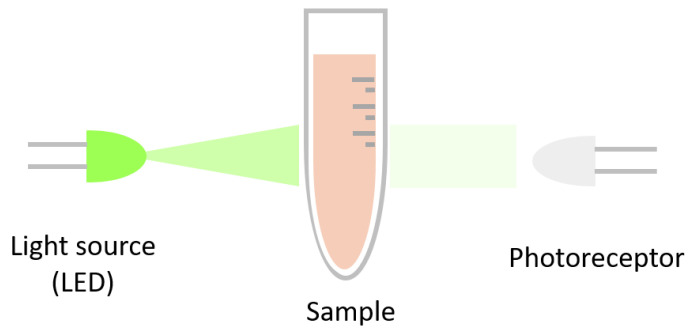
Absorbance mechanism: The light passes through the sample and specific wavelengths are absorbed by the molecules within it. Any light that is not absorbed continues its trajectory and is detected by a photodetector.

As light passes through the sample, specific wavelengths are absorbed by the molecules within it (see Figure 1). The amount of light absorbed depends on the properties of the substances and their concentration within the sample. Any light that is not absorbed continues its trajectory and is detected by a device, such as a photomultiplier, which quantifies the amount of light transmitted through the sample.

Absorbance is calculated using the Beer–Lambert law formula, which relates the intensity of the incident light $I_{0}$ and the intensity of the transmitted light *I*, as follows:

(1) $$A=\log\left(\frac{I_{0}}{I}\right)$$

This formula allows for the determination of the absorbance of the sample, which reflects how much of the original light has been absorbed by the molecules present in the sample.

Additionally, absorbance can be related to the concentration of the absorbing substance in the sample using the Beer–Lambert equation, as follows:

(2) A=ϵ·c·l

In this equation, ϵ is the molar extinction coefficient of the substance, c is the concentration of the substance in the sample, and *l* is the path length of the light through the sample. This relationship enables the calculation of the concentration of the substance in the sample using the measured absorbance, if the molar extinction coefficient and path length are known.

Absorbance measurements in lab-on-a-chip devices are a common technique for performing biological analyses. Traditionally, the need for a clear optical path between the emitter and the photodetector necessitated the use of glass as the fabrication material. The most direct method for analyzing byproducts via absorbance employed a transverse light path, where light crosses the microchannel, an approach adapted from conventional laboratory experiments using cuvettes.

The use of microdevices, however, entails a significant reduction in sample volume, which in turn diminishes performance due to the smaller quantity of liquid traversed by the light. To address this limitation, a planar configuration was first proposed nearly 30 years ago, in 1996 [29]. Depending on the orientation of the light path relative to the detection system, the setup is referred to as either planar or transverse. This work presents a microfabricated planar absorbance cell for integrated capillary electrophoresis devices. The device, made of glass, incorporates optical fibers, lasers, and photomultiplier tubes. The authors demonstrated improved performance thanks to the planar configuration, specifically due to its increased light path length.

Currently, the planar absorbance method is still in use, as seen in the work reported in [30]. In this example, the material is not glass but polymethyl methacrylate (PMMA), due to the increasing use of polymers. The device is designed for nitrite detection, but can also be applied to other analytes. Optical fibers are a good choice for focusing light on a microchannel section, guiding it to the detector, and enhancing measurement performance. However, they introduce minor challenges, such as a slight increase in the complexity of the microfluidic circuit, the need for precise fiber-to-microchannel alignment, and additional steps for assembling and disassembling the microfluidic chip.

The transverse method is still in use today, as demonstrated in the work reported in [31]. As can be observed in this example, the device is fabricated using the same mass-production material (PMMA). The authors developed a colorimetric ELISA-based point-of-care detection system for a lab-on-a-disc. This device does not include optical fibers, and its microfluidic chip insertion mechanism is user-friendly. A similar method is used in [6]; however, both the material and the reader differ. In this last case, PDMS serves as the primary material.

It is important to highlight that, regardless of the specific method or material employed, all these techniques share two consistent elements: transparent materials and an external electronics module. Section 6 will provide a comprehensive analysis of these shared characteristics and address several related important aspects.

The use of conventional printed circuit boards as substrate materials may appear to be a step backward technologically, due to their opacity; however, they offer important advantages, such as their potential for integration with electronic components. Like silicon, PCBs are not transparent, which necessitates the development of strategies to overcome their opacity, such as the one presented in [32]. It is worth noting that, in this example, the microfluidic part is made of PDMS, but it is placed on a micromachined silicon substrate with tilted walls designed to guide light from the LED to the photodiode.

In the context of PCB-based lab-on-a-chip devices, various methods have been employed to perform absorbance measurements. These methods will be analyzed below, focusing on aspects such as the system configuration, the selection of electronic components, and the integration of polymers. The most common approach involves placing an LED opposite a photoreceptor, allowing the liquid or sample undergoing the color change to pass between them.

**Figure 2:**
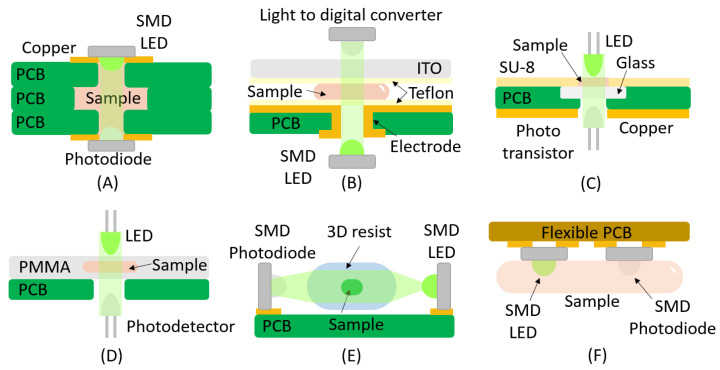
This figure shows several representative examples of absorbance detection systems implemented in Lab-on-PCB platforms. (**A**) Multiple stacked boards with integrated LED and photodiode; (**B**) structure using a plated-through hole into the PCB to create the light path; (**C**) structure using non-plated-through holes; (**D**) PCB-based lab-on-a-chip using non-plated-through holes to create the light path; (**E**) horizontal light path parallel to the PCB substrate developed right-angle SMD devices; (**F**) emitter and receptor are soldered using the conventional method, i.e., as conventional SMD devices.

In the first example (Figure 2A), it is demonstrated how the PCB can serve as the substrate for fabricating the microchannel, utilizing multiple stacked boards. It also supports the integration of the optoelectronic elements: the LED is soldered onto the top PCB, while the photodiode is mounted on the bottom PCB, as can be seen in [33] (see Figure 2A). This device works as a microfluidic pH regulation system based on printed circuit board technology, utilizing phenol red as a color indicator for optical pH monitoring by absorbance. A similar idea was reported by [34] for the detection of Fe^3+^ ions.

A similar approach was outlined in [35] (Figure 2B), using a surface-mounted device (SMD) LED and a SMD photodiode, which resulted in a digital microfluidic PCB-based platform for colorimetric nitrite sensing. Unlike the previous design, optoelectronic components were not directly integrated onto the PCB; instead, a plated-through hole was incorporated into the PCB to create the light path and compensate for the lack of transparency. Additionally, the microchannel consisted of an indium tin oxide (ITO) upper layer, with the PCB serving as the substrate, ensuring transparency throughout the rest of the device (see Figure 2B). A comparable configuration was used in a microfluidic platform for glucose detection [36] (Figure 2C); however, in this case, non-plated-through holes were employed to create the light path. In this work, through-hole (THT) LEDs are used, and a phototransistor replaces the photodiode as the photoreceptor. Building on this design, an advanced PCB-based lab-on-a-chip for glucose sensing was developed [37] (see Figure 2D).

The use of polymeric layers to fabricate microfluidic circuits on PCB substrates, coupled with the ability to integrate electronic components through copper tracks, allows for the soldering of right-angle SMD devices. This approach enables a horizontal light path parallel to the PCB substrate, as demonstrated in the study presented in [38] (see Figure 2E). The device described is a T-junction emulsion generator that employs an SMD LED and a phototransistor to detect the generated microparticles. In this system, the microfluidic circuit was manufactured using three-dimensional printing (3D-printed) onto the PCB substrate. Furthermore, this study explores various designs of 3D-printed lenses intended to focus the light from the LED onto the phototransistor.

An alternative method for positioning the LED and photodetector (phototransistor) is presented in [39] (see Figure 2F). In this case, the emitter and receptor are soldered using the conventional method, i.e., as conventional SMD devices. Although there is no direct light path, it is sufficient for monitoring the heart rate. Additionally, a flexible PCB is used, with nanocellulose as the substrate and inkjet-printed palladium (Pd) catalyst ink as the conductive material.

All of these examples demonstrate the versatility and high level of integration between polymers and electronic components enabled by PCB technology.

## 3. Fluorescence

Fluorescence is a phenomenon in which a substance absorbs light at a specific wavelength, typically within the ultraviolet or blue range, and subsequently emits light at a longer wavelength, such as in the green range [40,41]. In fluorescence measurement, a sample is excited by light of a specific wavelength, and the emitted light is then detected in order to quantify the concentrations of various components in the sample. The effect of varying the location of the blue LED will be analyzed in this section, as demonstrated in the provided examples. This technique is extensively employed in diagnostic and analytical applications due to its high level of sensitivity and capability to detect low concentrations of substances.

**Figure 3:**
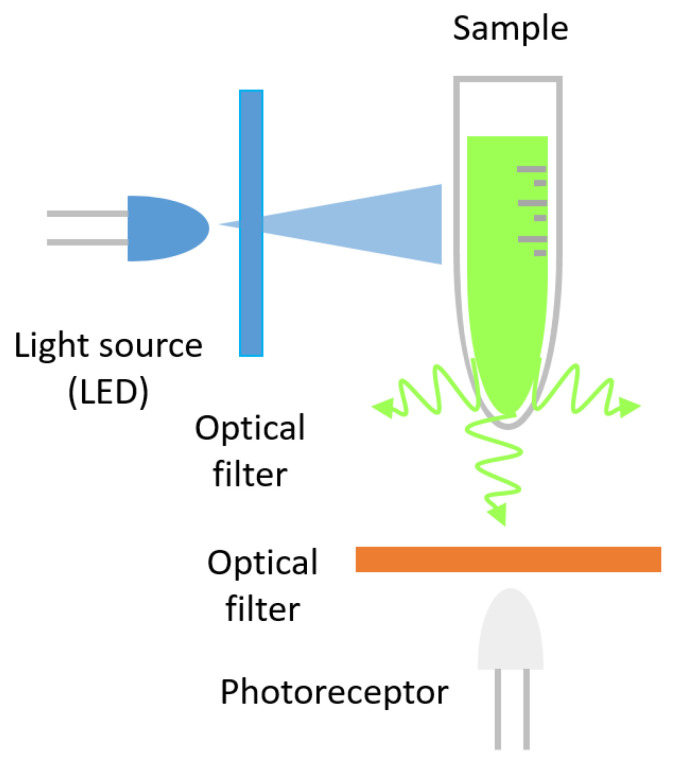
Schematic representation of the fluorescence process. The process consists of two main stages: excitation and emission.

The fluorescence process consists of two main stages: excitation and emission. During the excitation stage, a lamp or laser emits light at a specific wavelength, which is absorbed by the fluorophores in the sample. This absorbed energy excites the electrons of the fluorophores, elevating them to a higher energy state. As the fluorophores return to their lower energy state, they emit light at a longer wavelength, which constitutes the fluorescence that is subsequently measured (see Figure 3).

Fluorescence measurement relies on the intensity of emitted light, which is directly proportional to the concentration of the fluorescent substance in the sample. In contrast to absorbance, fluorescence intensity $I_{F}$ is influenced by the specific spectrofluorometer used, which means it is not a universal property. The relationship between fluorescence intensity and sample concentration is described by Equation [42], as follows:

(3) $$I_{F}=A_{0}\Phi_{f}\left(1-10^{-\epsilon cl}\right)$$

where $A_{0}$ is a machine constant (calibration factor of the instrument), $\Phi_{f}$ is the fluorescence quantum yield, ϵ is the molar extinction coefficient, *c* is the concentration, and *l* is the optical path length.

To analyze the sample, the emitted light is measured over a range of wavelengths, generating an emission spectrum. Each fluorophore exhibits a unique spectral profile, enabling the identification of different components within the sample. For quantitative analysis, the intensity of emitted light at a specific wavelength is compared to a calibration curve previously established for each analyte. Additionally, optical filters are essential due to the close overlap between the excitation and emitted light wavelengths.

In fluorescence measurements, it is crucial to account for the phenomenon of quenching, which reduces fluorescence emission. Quenching occurs when the sample contains either high concentrations of fluorophores or specific compounds that interfere with the fluorescence process.

Focusing on lab-on-a-chip systems that incorporate fluorescence detection, the work reported in [43] presents different configurations, based on external excitation and imaging, with filters, dichroic mirrors, light sources, and photodetectors placed outside the lab-on-a-chip system. The work demonstrates improvements in both in-line and off-line configurations, when appropriate components are selected. The authors of [43] described a cost-effective device in which the excitation filter and dichroic mirror can be eliminated when the light source and detector are carefully selected. For example, the narrow spectrum of laser light can eliminate the need for an emission filter. Other techniques, such as the use of polymer optical fibers, have also been employed to focus blue light for fluorescence applications [44]. In addition, the authors of [43] described important issues for polymers. Among other technical comments, the authors commented that, when developing LoC systems designed for fluorescence, materials must be transparent in the visible range and exhibit as little autofluorescence as possible to minimize background signals. These devices could be fabricated using transparent materials like glass or polymers such as PDMS or SU-8. These materials could be only used as the top cover, and the rest of the chip was made using opaque materials. Among possible transparent materials, cyclic olefin copolymer (COC), borosilicate glass, and PDMS, show low autofluorescence, while SU-8 and PMMA produce higher background signals. To improve fluorescence detection, light ranging between 635 and 650 nm is preferred. Additionally, beyond the sensitivity of photodetectors, signal conditioning plays an important role by amplifying the detected signals and minimizing noise to achieve the desirable high signal-to-noise ratio.

Moreover, fluorescence has been also applied in droplet microfluidics, as described in [45]. The OptiDrop platform, reported in this work, utilizes a measurement method based on optical detection with photomultiplier tubes (PMTs), which capture scattering and fluorescence signals generated by a single blue laser (488 nm) with high sensitivity. The technology is integrated into a microfluidic chip with optical fibers. Its effectiveness was demonstrated in detecting the Major Histocompatibility Complex (MHC), highlighting its applicability in research and diagnostics.

Finally, ongoing studies aim to enhance the guidance available on this path. For example, the work reported in [46] focused on fluorescence excitation. In this paper, an integrated glass microprism matrix for light coupling was proposed. The authors described the fabrication process of the glass microprism arrays based on silicon wafers. These structures were combined with microfluidics and designed to allow laser beam coupling into the substrate.

Regarding PCB technology applied to fluorescence detection for lab-on-a-chip applications, the developed devices show similarities with the ones for absorbance. The following section examines various fluorescence detection systems implemented with PCB technology. These systems vary in aspects such as the type of components chosen, their positioning or integration approach, and the inclusion of different materials or supplementary components.

**Figure 4:**
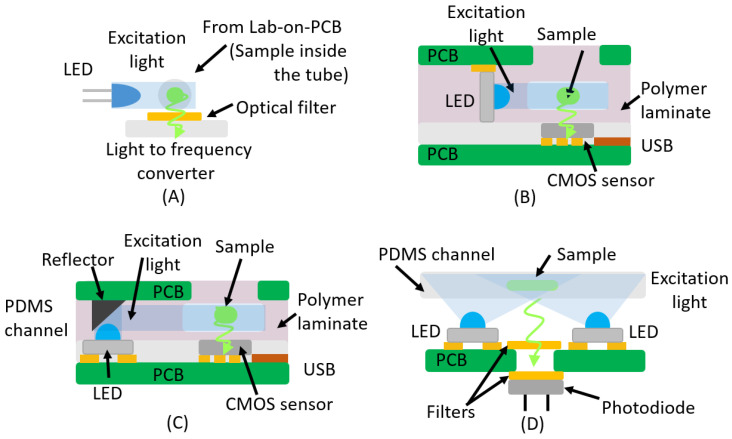
This figure presents a selection of fluorescence detection systems implemented in Lab-on-PCB platforms that have been examined in this review. The sample emission is illustrated by green rays, while the excitation light is depicted as a blue light beam. (**A**) The LoCKAmp platform utilized a blue through-hole LED as an excitation source while fluorescence emission was captured by a light-to-frequency sensor; (**B**) the blue SMD LED is soldered in alignment with the PCB, ensuring that its light remains parallel to the board; (**C**) the blue LED is integrated so that the light is perpendicular to the PCB. Therefore, the authors had to integrate a reflector onto the PCB to guide the light path parallel to the PCB; (**D**) the LEDs and photodetector are positioned perpendicularly to the PCB to optimize detection efficiency.

For example, the most direct solution is the use of a through-hole LED, as proposed in [16,47] (see Figure 4A). The authors developed the LoCKAmp system and evaluated its effectiveness at detecting SARS-CoV-2 across two critical scenarios: clinical nasopharyngeal specimens and pre-processed wastewater samples. The platform utilized a blue through-hole LED as an excitation source for the fluorescent dye (GelGreen^®^), while fluorescence emission near 535 nm was captured by a light-to-frequency sensor governed by a custom Arduino-based circuit. To enhance signal clarity, a cost-effective commercial optical filter was positioned above the detector, preventing interference from the excitation LED. Moreover, the LoCKAmp lab-on-PCB chips were produced in an ISO-certified PCB-manufacturing facility, with small-scale production costs estimated at £2.50 per chip in 2023 (Figure 4A).

Other more complex options can be found in the literature, including the integration of additional electronic components or the use of different structures. For instance, the method employed in isotachophoresis applications offers a notable example [48] (Figure 4B). This technique, in analytical chemistry, is used for the selective separation and concentration of ionic analytes. As a form of electrophoresis, it separates charged analytes based on their ionic mobility. The device consists of a reader PCB cassette, which houses the CMOS sensor, and a disposable microfluidic PCB cartridge that integrates a microfluidic channel, a blue SMD LED, and high-voltage electrodes. The blue SMD LED is soldered in alignment with the PCB, ensuring that its light remains parallel to the board (Figure 4B). This PCB-based device performs isotachophoretic separation and fluorescence detection. A similar microfluidic PCB-based device for lab-on-a-chip fluorescence detection was reported by the same authors [49,50]. The structure of the device follows the same general idea (Figure 4C). However, in this case, the blue SMD LED is integrated so that the light is perpendicular to the PCB. Therefore, the authors had to integrate a reflector onto the PCB to guide the light path parallel to the PCB and along the microfluidic channel (Figure 4C). This solution is less interesting than the one previously discussed, due the integration of the reflector.

The excitation light can be configured as well, in different ways, as demonstrated in the setup reported in [51] (Figure 4D). This work presented a portable, low-cost fluorescence sensing system for the on-site detection and quantification of microalgae samples. The system utilizes multiple blue LEDs (peak wavelength: 448 nm) for excitation and a silicon photodiode to measure the fluorescence emitted by chlorophyll a. The LEDs and photodetector are positioned perpendicularly to the PCB to optimize detection efficiency. A dichroic mirror filter and a color filter ensure that only the fluorescence signal from chlorophyll a reaches the photodetector (Figure 4D). Additionally, the system incorporates a PDMS microfluidic chip to hold the sample, with the photodiode placed beneath the chamber to detect the emitted light. Optical filters above and below the PCB minimize noise, with the dichroic filter acting as a low-pass filter for 647 nm light and the color filter serving as a low-pass filter for 650 nm fluorescence.

**Figure 5:**
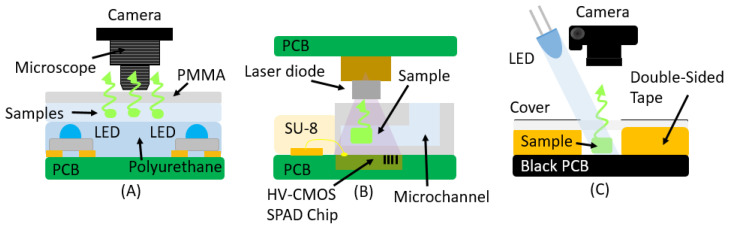
In this image, more advanced examples are depicted. (**A**) PCB-based microfluidic prototype with blue SMD LEDs, flow with fluorescent microbeads, and detection using an inverted microscope; (**B**) semiconductor single-photon avalanche diode (HV-CMOS SPAD) detector array embedded on a PCB; (**C**) black-coated PCB-based micro-PCR.

However, the use of this detection technique with PCB substrates has led to the detection of a phenomenon that must be considered: autofluorescence. This interesting characteristic of flame retardant 4 (FR4) was observed by [52]; that is, it emits autofluorescence under blue light. The authors developed a PCB-based microfluidic prototype using 1002F and polyurethane with integrated resistors, temperature sensors, and blue SMD LEDs for on-board thermal control, detection, and optical imaging. To minimize FR4 autofluorescence, observed under blue light, the platform included a machined window. On-chip lighting control was demonstrated by imaging sodium dodecyl sulfate (SDS) buffer flow with fluorescent microbeads, using only the integrated SMD LEDs and an inverted microscope (Figure 5A).

An alternative excitation method utilizes laser diodes (Figure 5B). In this context, the device reported in [53] implements fluorescence lifetime spectroscopy for molecular diagnostics, integrating a 405 nm ultraviolet laser diode as a pulsed excitation source and an ultra-sensitive high-voltage complementary metal–oxide–semiconductor single-photon avalanche diode (HV-CMOS SPAD) detector array embedded on a PCB (Figure 5B). By measuring fluorescence decay after the excitation pulse fades, this approach eliminates the need for optical filters, making it particularly suitable for long-lifetime fluorophores. The device achieves high sensitivity comparable to conventional optical instruments, enabling molecular diagnostics from small sample volumes at practical concentrations. The elimination of optical fibers, combined with the use of PDMS as a transparent material, presents an interesting approach. PDMS was chosen for microfluidics due to its excellent optical properties across the near-ultraviolet, visible, and near-infrared (NUV-VIS-NIR) spectrum (400–1000 nm). Unlike FR4, PDMS exhibits minimal autofluorescence, further improving detection sensitivity.

Regarding the effects of blue light on PCBs, the study reported in [54] adopted an innovative approach (Figure 5C). To optimize fluorescence detection, the researchers developed a black-coated PCB-based micro-polymerase-chain-reaction (micro-PCR) chip, designed to minimize noise caused by light reflection. Traditional green PCBs reflect blue LED light, reducing the signal-to-noise ratio (SNR), whereas the black solder mask effectively mitigates this interference. Additionally, the reaction chamber area was printed with white silk to enhance detection accuracy. The chip was assembled using commercial adhesive tapes and plastic covers, and six different reaction chamber designs were tested. Fluorescence imaging was performed by briefly illuminating the amplified product with a blue LED before capturing the signal, turning the LED off immediately afterward to prevent probe quenching (Figure 5C). A Canon 1100 Digital Single-Lens Reflex (DSLR) camera (Canon Inc., Tokyo, Japan) was used to record fluorescein amidite (FAM) fluorescence signals with improved clarity.

As shown herein, the materials are not only the issue to take into account; the color of the materials is also very important for this detection method, as will be commented on Section 6. Moreover, the effect of certain specific characteristics, such as autofluorescence or quenching, must be taken into account.

## 4. Chemiluminescence

**Figure 6:**
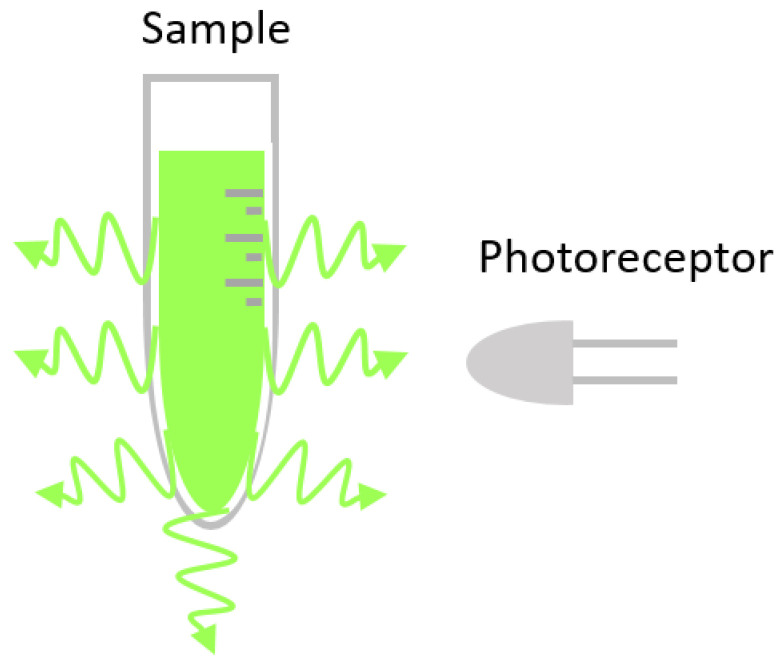
Chemiluminescence mechanism: the light is emitted as a result of a chemical reaction and detected using an optical device.

Chemiluminescence is a phenomenon in which light is emitted as a result of a chemical reaction. This technique is widely employed in biosensors due to its inherent advantage of not requiring an external light source, as the light is produced directly by the chemical reaction (see Figure 6).

Typically, it involves a substrate reacting with an enzyme or another chemical compound to generate light. The intensity of the emitted light is directly proportional to the analyte concentration in the sample, allowing its concentration to be inferred by measuring the light intensity. The reaction produces a product in an electronically excited state, which subsequently releases visible light as it returns to its ground state. To provide the necessary energy for this process, the reaction must be sufficiently exothermic, which is why strong oxidizing agents such as oxygen, hydrogen peroxide, or other reactive species are commonly used. In general, at least two reactants (A and B) combine to form a product (C), which may exist in an excited state $C^{*}$. As it transitions back to its ground state, it emits a photon, producing the characteristic light emission [55,56]:

(4) $$A+B\to C^{*}\to C+h\nu$$

where *A* and *B* are the reactants involved in the chemical reaction, *C* is the resulting product, $C^{*}$ denotes the electronically excited state of the product, and hν represents the photon emitted as the excited product returning to its ground state.

There are many subsets of chemiluminescence, including, bioluminescence in living organisms [57], electrochemiluminescence [58], thermochemiluminescence [59], sonoluminescence [60,61], crystallochemiluminescence [62], and radioluminescence [63].

Since chemiluminescence does not require an external light source for excitation, the only electronic component needed for detection is the sensor. In this regard, various types of detectors have been employed for chemiluminescence-based measurements [4,64]. Many of these detectors are compatible with fluorescence-based detection.

Typical lab-on-a-chip systems for chemiluminescence incorporate P-type, intrinsic, and N-type (PIN) photodiodes [65], single-photon avalanche diodes (SPADs), photomultiplier tubes (PMTs), or a combination of the latter two [66]. In addition, charge-coupled devices (CCDs) [67] and complementary metal–oxide–semiconductor (CMOS) sensors [68] are also used as photodetectors for chemiluminescence.

In the context of PCB-based chemiluminescence devices, the literature reports various implementations involving different types of detectors, additional components, and the integration of materials with distinct characteristics.

**Figure 7:**
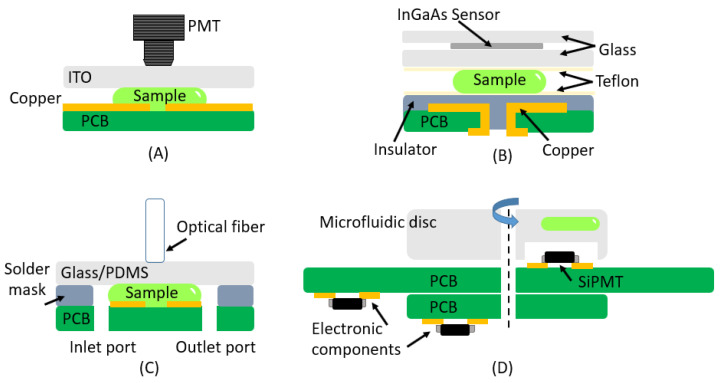
This image displays several examples of chemiluminescence-based detection systems implemented on PCBs. (**A**) PMT placed above a PCB-based digital microfluidics cartridge, enabling precise detection; (**B**) the system integrated a thin-film InGaAs metal–semiconductor–metal photodetector; (**C**) the system includes gold microelectrodes, the solder mask acting as a spacer and the microfluidic circuit fabricated using PDMS; (**D**) platform based on a modular PCB design, where system-specific components are integrated into the main PCB.

The most commonly used detector is the PMT, which is shared across the entire lab-on-a-chip system for detecting similar effects. For example, the study reported in [69] utilized a PMT placed above a PCB-based digital microfluidics cartridge, enabling precise chemiluminescence detection, as shown in Figure 7A. This approach was employed to detect B-type natriuretic peptide (BNP) analytes. The system utilizes magnetic beads for the immunoassay, while the PCB, together with a polyethylene (PE) dielectric layer, enhances droplet control through electrowetting, which is critical for the cartridge’s functionality. A similar structure was developed in [70].

There are approaches which enable the substitution of PMTs for other optical sensors, such as that used in [71,72] (Figure 7B). In this work, a digital microfluidic system based on a PCB was developed. The system integrated a thin-film InGaAs metal–semiconductor–metal photodetector. This sensor was attached to the top glass plate, coated with Teflon AF (amorphous fluoropolymer), enabling optical detection within the system. Its functionality was evaluated through the manipulation and mixing of chemiluminescent droplets, confirming its ability to detect the resulting emission (Figure 7B).

PCB-based devices are a suitable choice for electrochemiluminescence due to the requirement of an electric field. The copper layer of the PCB can be used to fabricate the electrodes, and gold can be added if needed. These electrodes generate the required electric field. An illustrative example of this can be seen in Figure 7C, which shows the device developed in [73] integrates electrochemiluminescence analysis in microfluidic systems using PCB technology. The system includes gold microelectrodes, with one serving as the working electrode and the other as the reference electrode; the latter is coated with a thin film of Ag/AgCl. Additionally, the solder mask acts as a spacer. The microfluidic circuit is fabricated using PDMS. Electrochemiluminescence signals are collected via an optical fiber positioned near the working electrode surface. The signal is then transmitted through a liquid-core optical fiber and detected by a photomultiplier tube-based luminometer. A similar method was employed in [74], with the addition of a compact CMOS Buried Double p-n Junction (BDJ) photodetector module.

PCB technology is also compatible with other technologies, such as silicon photomultipliers (SiPMs), providing an cost-effective solution for optical detection. In this respect, the study reported in [75] developed a platform based on a modular PCB design, where system-specific components are integrated into the main PCB (Figure 7D). This approach allows micro-electro-mechanical sensors (MEMS), such as the SiPM, to be positioned directly next to the mixing chamber, eliminating the need for complex optical setups to guide light from the sample to the sensor. Additionally, Ref. [75] provides a comprehensive comparison of the key characteristics of various photodetectors used for chemiluminescence detection.

## 5. Other PCB-Based Methods

After analyzing in the previous sections the most commonly used phenomena in optical detection, absorbance, fluorescence, and chemiluminescence, we will now discuss several less common PCB-based detection methods and techniques. In addition, their compatibility with conventional PCB substrates will be discussed.

Regarding the most typical optical methods used in lab-on-a-chip systems, the evanescent wave phenomenon is the most important one. Evanescent wave phenomena refer to optical effects that occur when an electromagnetic wave undergoes total internal reflection at an interface, generating a non-propagating field that decays exponentially into the adjacent medium. These waves can interact with nearby materials, enabling applications in sensing.

**Figure 8:**
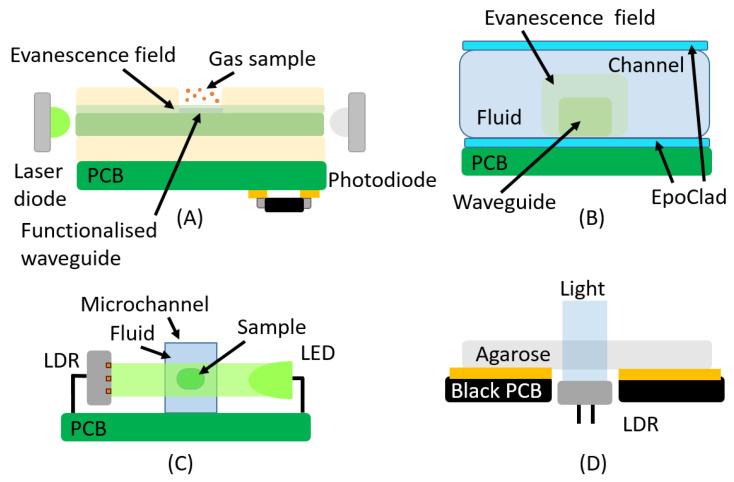
Two examples of systems employing the evanescent field and two integrating LDRs are presented in this case. (**A**) PCB-integrated ammonia gas sensor based on evanescent wave detection; (**B**) the absorbance occurs through the interaction between the evanescent field and the sample; (**C**) the system employs a green LED and an LDR to measure voltage variations as droplets pass through a fluidic channel; (**D**) the LDR was placed beneath a hole in the PCB to measure turbidity.

This technique has intrinsic characteristics and material and configuration requirements that pose challenges for PCB technology; however, the creation of evanescence waves is possible on PCBs. Some implementations have been achieved; for instance, the sensor reported in [76] (Figure 8A) showed a PCB-integrated ammonia gas sensor based on evanescent wave detection. In order to do so, multimode polymer waveguides, functionalized with chemical dyes, were directly fabricated on the underside of the PCB, while all electronic components were placed on the top side, as shown in Figure 8A. The optical absorption of these dyes changed in the presence of ammonia, enabling detection in both gas and liquid phases. The sensor employed 635 nm laser diodes as light sources and AlGaAs/GaAs photodiodes as detectors. The evanescent field interacted with the dye in exposed waveguide regions, causing optical attenuation changes proportional to the ammonia concentration. The integration of multiple waveguides with different dyes enabled the simultaneous detection of various substances within a single device.

The integration of polymeric waveguides on PCBs is a promising approach for enhancing the functionality and versatility of PCB-based devices, as can be seen in the following works [77,78,79,80,81], where different strategies and methods of fabrications have been carried out. One of the fabrication process involves laser direct writing on SU-8 to define the future waveguides [79] (Figure 8B). The authors demonstrated that this method can also be used for absorption-based applications [82]. However, this is not a direct absorbance measurement, as in Section 2, where the light passes through the liquid sample. Instead, the absorbance occurs through the interaction between the evanescent field and the sample, causing the attenuation of the guided light. Surface-mounted device LEDs and photodiodes were integrated into their right-angled version, as shown in [38].

Another technique related to evanescent wave phenomena is surface plasmon resonance (SPR) [83,84]. This technique is highly sensitive to changes in the refractive index near the surface, enabling the real-time detection of molecular interactions. In SPR, a polarized light beam is directed onto a metal film at a specific angle of incidence. When the angle and wavelength of the light match the resonance condition, surface plasmons are excited, leading to a decrease in the reflected light intensity. The shift in the resonance angle or wavelength is then used to analyze binding events at the surface. Generally, the thicknesses of metal films for sensors range from tens to hundreds of nm; the typical thickness for a gold film, for example, is around 50–100 nm [84,85]. This thickness is important for optimizing the sensitivity of SPR measurements.

The surface plasmon resonance method has been successfully integrated into lab-on-a-chip systems [83]. However, to date, no application involving SPR has been implemented using PCB technology. Although a gold layer is available in commercial PCBs, the previously discussed thickness, structural configuration, and integration with FR4 substrates, which are commonly used as a substrate in PCBs, pose significant challenges for reproducing the conditions required for conventional SPR-based sensing.

This is also the case for a different configuration of SPR devices, namely localized SPR (LSPR). This phenomenon has been the subject of great scientific interest in recent years as a novel counterpart to the well-established SPR sensor. In this method, the gold membrane is typically replaced by gold nanoparticles [86,87,88,89,90]. Similarly to conventional SPR methods, LSPR also requires transparent materials. This poses a limitation for PCB technology, which can be addressed through the integration of transparent materials such as PDMS, PMMA, and ITO, among others. Regarding the LSPR configuration and PCB technology, the integration of nanoparticles is compatible with printed circuit boards. Regarding the materials, commercial polyethylene terephthalate (PET)-based PCBs represent a promising alternative due to their transparency and flexibility. Similar approaches have been successfully integrated into lab-on-a-chip systems using alternative materials, such as a PDMS–glass–gold composite [91] and polymers [92], but not yet with PCB technology.

Regarding optical sensors typically used for PCB applications, light-dependent resistors (LDRs) are a common type that have seen limited use in biomedical applications. In this context, the study reported in [93] proposed a low-cost optical droplet sensor for characterizing droplets in Lab-on-a-PCB devices (Figure 8C). The sensor employs a green LED and an LDR to measure voltage variations as droplets pass through a fluidic channel. It was calibrated to detect and characterize various multiphase flow parameters, such as velocity, flow rate, droplet length, and volume. A different biomedical application involving an LDR was reported in [94] (see Figure 8D). This work described a Lab-on-PCB system for agarose gel preparation and electrophoresis. In this case, the LDR was placed beneath a hole in the PCB to measure turbidity during the gel preparation process, enabling the detection of the endpoint of agarose curing. White light was used as the illumination source, and the PCB’s solder mask was black (Figure 8D). It is worth noting that, although LDRs in PCB applications are typically used to measure opacity, most of them exhibit a spectral response in the range of 525–550 nm, which corresponds to green light.

## 6. Discussion

Taking into account the phenomena discussed in this review, the implications of each regarding the use of PCB technologies are analyzed. Moreover, the high-level integration with materials, specific characteristics of PCBs, and the selection of particular optical elements will also be discussed.

As previously mentioned, the most common optical methods used in PCB-based lab-on-a-chip systems are absorbance, fluorescence, and chemiluminescence. These techniques vary in terms of complexity regarding the required optoelectronic components and material transparency. Absorbance and fluorescence, for instance, require a clear optical path between the light source and the photodetector, necessitating the use of transparent materials along this path, as well as both emitter and detector components. In contrast, chemiluminescence does not require an external light source, since the sample emits light as a result of the chemical reaction. Given that PCBs are not transparent, different strategies can be implemented to enable optical detection, such as integrating transparent materials like PDMS, PMMA, or ITO. Regarding sensitivity, absorbance detection is typically the least sensitive, followed by fluorescence; finally, chemiluminescence shows the highest sensitivity. In terms of cost, absorbance systems are the most affordable. Fluorescence detection systems are moderately expensive, and chemiluminescence setups, especially those involving enhanced signal detection, are typically the most expensive.

Regarding the materials integrated into PCBs, transparent materials such as glass, PMMA, and PDMS are the most commonly used. These materials are well-suited for optical detection. However, thermoplastics are increasingly being adopted instead of glass, as the manufacturing methods for glass devices are more demanding and complex. Furthermore, polymers are less expensive, easier to process using mass production techniques such as hot embossing, and significantly less brittle, making them a more practical choice for robust devices and large-scale production. Another material mentioned in the paper is Teflon AF, which is valuable for applications requiring transparency, minimal optical interaction, and stability. Alternatives such as ITO, as a substrate material, or SU-8, as a microfluidic circuit or waveguide, also could be used. However, ITO is a very expensive material, which contrasts with the low-cost nature of PCB technology. Additionally, SU-8 is a yellowish material that absorbs light at low wavelengths [95]. Regarding commercially available PCB materials for optical applications, PET substrates are an interesting option to explore.

Regarding the specific characteristics of PCBs, when designing PCB-based optical devices for fluorescence, it is necessary to consider the optical properties of a printed circuit board. As previously mentioned, the choice of solder mask can have an impact, as the FR4 substrate and green solder mask may produce autofluorescence; however, this effect is minimized with a black solder mask. Additionally, the use of a white silk mask could enhance measurement performance. When selecting polymer materials for the microfluidic circuit on a PCB-based substrate, the potential for autofluorescence must also be considered [96]. However, in fluorescence detection, the fluorophore is the key factor, as it is what actually emits the fluorescent signal. Nevertheless, the color of the material can influence detection efficiency by adding background noise.

Additionally, another specific characteristic of PCBs is their surface finish. The authors of [97] suggested that adding a copper plane in specific areas of the printed circuit board, alongside the electrodes, improves observation. This copper plane should not be connected to the electrodes. It is recommended to use a hot air solder leveling (HASL) surface finish and omit the solder mask, thereby creating a reflective surface. This approach enhances cell visibility under a microscope when a top-lighting source is used.

Finally, regarding the light source, the narrow spectrum of laser diodes not only enables the use of a more precise and efficient light source, but also simplifies the design and operation of optical systems by eliminating the need for additional components, such as emission filters, typically used to block out-of-band light.

A Table 1 is shown below that summarizes the optical detection phenomena explained throughout this article, along with their distinguishing characteristics, the electronic components involved, and the related references.

## 7. Conclusions

Lab-on-PCB technology has proven to be a highly versatile platform for optical sensing, enabling compact, cost-effective, and integrated detection systems. Through the analysis of different optical detection methods, including absorbance, fluorescence, chemiluminescence, and evanescent wave sensing, it is evident that each technique presents distinct advantages and limitations when implemented on PCB substrates.

From the reviewed works, LEDs are the most frequently used excitation sources across all detection methods due to their low cost, small footprint, and ease of integration into PCB designs. Laser diodes, although less common, are primarily used in fluorescence and evanescent wave applications, where coherent and high-intensity light sources are required. Chemiluminescence, in contrast, does not require an external light source, relying instead on chemical reactions to generate detectable signals.

Regarding photodetectors, photodiodes are the most widely employed due to their compatibility with PCB-based electronics, followed by CMOS sensors, which offer higher sensitivity and spatial resolution, particularly in fluorescence detection. Phototransistors and PMTs appear in specific cases where enhanced signal amplification is needed.

The integration of electronic components is one of the key strengths of LoP technology, as demonstrated in the examples presented in this article. The majority of the reviewed studies have successfully incorporated LED-photodiode pairs directly onto PCBs, demonstrating a high degree of miniaturization and integration. Fluorescence-based systems, while increasingly integrated, still often rely on external optical components such as filters and lenses, which pose challenges for full PCB-based implementation. Evanescent wave sensors represent a promising approach for achieving highly integrated optical detection directly on PCB substrates.

Overall, PCB-based optical detection systems continue to evolve, benefiting from advancements in component miniaturization and fabrication techniques. Future work should focus on further enhancing integration levels, improving sensitivity, and exploring new optical sensing strategies that leverage the unique properties of PCB technology.

Finally, based on our experience and the needs identified over the years, both optical integration for biomedical applications and PCB systems in general could benefit from several interesting improvements. For example, fully transparent PCBs could be useful, i.e., wherein both the dielectric and the conductor are transparent. By adopting the characteristics of well-known MEMS substrates, the development of cavity-PCBs, inspired by the concept of cavity-silicon-on-insulator (c-SOI), could expand device development possibilities thanks to the manufacturing opportunities offered by the cavity. Furthermore, expanding the range of options for the PCB conductor, which is typically made of copper, by using metallic or polymeric materials with piezoelectric or piezoresistive properties could boost the development of PCB-based sensors and actuators.

## Figures and Tables

**Table 1:** PCB-based devices with optical sensing methods.

Absorbance				
Excitation	Detection	Integrated	Characteristic	Reference
LED	Photodiode	Yes	SMD	[33,34]
LED	Photodiode	No	SMD	[35]
LED	Phototransistor	No	THT	[36]
LED	Photodetector	No	PCB Hole	[37]
LED	Photodiode	Yes	SMD Vertical/Lens	[38]
LED	Phototransistor	Yes	Flexible	[39]
**Fluorescence**				
**Excitation**	**Detection**	**Integrated**	**Characteristic**	**Reference**
LED	Light-to-frequency	No	THT LED	[16,47]
LED	CMOS Sensor	Yes	SMD/Reflector	[49,50]
LED	CMOS Sensor	Yes	SMD Vertical	[48]
LED	Photodiode	Yes	SMD/THT Perpendicular	[51]
LED	Inverted Microscope	Yes	Window	[52]
Laser diode	HV-CMOS SPAD	Yes	Embedded	[53]
LED	DSLR Camera	No	Black Solder Mask/White Silk	[54]
**Chemiluminiscence**				
**Excitation**	**Detection**	**Integrated**	**Characteristic**	**Reference**
N.A.	PMT	No	-	[69,70]
N.A.	InGaAs MSM detector	Yes	Teflon AF	[71,72]
N.A.	PMT	No	Optical Fiber	[73]
N.A.	PMT/CMOS BDJ	No	Optical Fiber	[74]
N.A.	SiPM	Yes	Modular	[75]
**Other Methods**				
**Excitation**	**Detection**	**Integrated**	**Characteristic**	**Reference**
Laser diode	AlGaAs/GaAs photodiodes	Yes	Polymer waveguides	[76]
LED	Photodiode	Yes	Polymer waveguides	[79]
Lamp	LDR	No	PCB Hole	[94]
LED	LDR	No	-	[93]

## Data Availability

Not applicable.
